# Annual Address of the Michigan Dental Association Delivered at Ann Arbor, Oct. 16, 1873

**Published:** 1874-07

**Authors:** G. R. Thomas


### DENTAL REGISTER.

VOL. XXVIII.] JULY, 1874. [No. 7.

### ANNUAL ADDRESS OF THE MICHIGAN DENTAL ASSOCIATION, DELIVERED AT ANN ARBOR, OCTOBER 16, 1873.

BY G. R. THOMAS, PRESIDENT.

Another year has been added to the history of our profession, since last we met, for an interchange of ideas, and to deliberate upon those subjects which are vital to the well-being of an ever afflicted people. And it becomes, according to custom, the duty of your presiding officer, to address you upon some topic of interest, if only to refresh our minds, by turning them back for a little to an already familiar channel. On such occasions, it is perhaps usual to enlarge upon the progress made by the profession in general, and to congratulate ourselves upon the giant strides made by it within the past thirty years; which is in itself something truly marvelous, and, possibly, we need the stimulus to urge us forward to encounter the many difficulties and perplexities incident to the thorough and conscientious practice of dentistry. We are as a profession taking a stand which gives us the respect of the civilized world; although our advancement thus far, has been through adverse circumstances whose name is legion, most gigantic and irrepressible, among them being false representations and practices of men who set themselves up to be something when they were *worse* than *nothing*. The gradual metamorphosis of dentistry, from being a part of the juggler's art, (who, promising to extract teeth without pain, had men stationed behind him to make at a given signal, a noise, so sudden and deafening, that in the patient's surprise and fright, he would not notice the pain of the operation) up to being a mere adjunct of the barber's trade; and farther on, till it is in the hands of men skillful and scientific, such as Koecker, Hudson, Hayden, Harris, Tomes and many others, who gave it the illumination of brilliant intellect and clear judgment, is scarcely *less* wonderful, than the transformation of the chrysalide, into the beautiful winged butterfly. We feel to-day that we justly merit something better than the contemptuous appellation of "tooth carpenters," and take for America the honor of elevating ours up to the level of liberal professions. In consideration of all that has been done in the past, and that our structure may grow in beauty, and symmetry, in the future, the dentist of today, should not be content to plod along in the paths marked out, even by acknowledged great men. The fields of knowledge, yet undiscovered and undeveloped, are many and vast, and should we fold our hands and say "We know enough" "There is no use in going further!" What philanthropic, whole souled man can look at an infant, already beginning to suffer from decayed teeth, even before all are erupted, without making an effort to discover some means to arrest, or still better to prevent all this suffering. We have seemingly reached almost the height of perfection in arresting dental caries, which is in itself a very important step, but very little has been accomplished, in any practicel way to prevent its appearance.

Is this the highest progress? Once in a while we hear of some earnest man making a decided stand in favor of a pet theory, and for a little time perhaps we are quite filled with the megnetism of his zeal. But alas, there is little, or no concert of action. In order to the accomplishment of solid and lasting good, we must work together, and with our forces combined, some great good must result. Very seldom do we see one man who can reduce a theory to practice without flying wide of his mark many times. But if we together take up this matter, we shall doubtless reach a definite and tangible result, the effect of which will be to materially lessen the sufferings of our fellow mortals. The first application of steam as a motive power, was rude and imperfect, compared with the many fine and beautifully wrought appliances which meet us at every turn, but many minds were necessary to bring to such perfection the original discovery. And history shows us that those epochs of advance which have particularly marked the nineteenth century, have been the result of concerted action. The different colors of light are beautifully brought out by the spectrum, and so by proper reflectors the light being thrown on any dark object it is immediately illuminated, our separate rays of light being converged upon the dark question of prevention of dental caries analyzed, reflected, and tested in association and convention, in time the hidden depths must be revealed and explored. Dental Surgery is just now taking up a new line of march heretofore untrod, and who would block the way to prevent its triumphs, all present will respond, not I, not I, and although we may not all be alike competent to instruct or lead in this onward march, still we can encourage others, and I venture the assertion that we feel alike the necessity of putting forth every energy to leave the profession better than we found it; how can we best accomplish this? Upon my first visit to a dentist's office, in the days of my boyhood, some twenty years ago, I well remember having seen, over the laboratory door in bold letters these words, "positively no admittance" and I have often thought since, that perhaps the fact of my having become connected with this profession, is due to the strange impression there made upon my mind. It was in my native town and I at once commenced to court favor with the doctor (not through any particular affection I had for him or his services) but simply that I might gain access to that mysterious laboratory. I was not long in accomplishing my purpose, nor much longer in ascertaining that the words over the door, were not so much for preventing the entrance of the simply curious lookers on, as to conceal what little light he possessed under a bushel, lest some one equally ambitious as himself, should become master of the secrets therein contained: years of practice have done much to relieve my mind of those first and imperfect impressions of the science of dental surgery, but I fear that a life long practice will not suffice to completely obliterate them. If the closed doors had served to keep out of the profession all, except such as would prepare themselves for an honorable place in its ranks, I would, (and so would we all) say amen, but such has not been the case; on the contrary, its tendency was (I speak of it in the past tense, for I believe it can truly be numbered among the things of the past) to actuate many to enter it purely from mercenary motives, believing it to be an easy and profitable way to gain a competency, and when a student did matriculate, it was for three, six months, or a year at longest.

Think of it gentlemen, a year in which to prepare an uncultivated mind to practice upon the vital and active tissues of the human body. I know not how many of the members of this association have students in your charge, but I would most respectfully call the attention of all such to the resolution adopted by this association, October 14, 1870, which may be found on the last page of the printed manuals containing the constitution and rules of this association and reads as follows:

RESOLUTION ADOPTED, OCTOBER 4, 1870.

"Whereas, this association is prepared to labor in the future, as in the past, for the elevation of dentistry to the standing and dignity of a learned profession.

And whereas, the practice of admitting persons into our offices to "learn dentistry" and allowing them to go out in a few weeks or months as dentists, without a moiety of the professional knowledge and skill a dental practitioner ought to possess, is unprofessional and injurious to the public whose health we profess to conserve, and in every way reprehensible.

Therefore, *Resolved*, that we the members of the Michigan Dental Association hereby pledge ourselves each to the others that we will not take, or allow any person in our offices as student, or learner, or working under instructions, unless such person shall pledge himself to study dentistry at least two years under the instruction of a reputable practitioner of dentistry and attend at least two full courses of lectures at a Dental College, and graduate before offering his services to the public as a dentist."

Therein is the key which may regulate our actions in this matter, but the love we bear the profession, the community whom we serve, as well as the student whose teacher we are, together with the proper exercise of good judgment and discretion, ought to render such a resolution quite unnecessary at our hands. Is there one here who can say, "I was prepared for the practice of the profession when I began?" Feeling this as we do, are we not better qualified, (if not to teach) to endeavor with might and main to stimulate those who are to succeed us, to attain to far brighter and nobler heights, than has ever been our lot to reach, no boy should be allowed to commence the study of dentistry simply because he is ingenious and has a natural taste for mechanism; true this should be a consideration, but by no means the qualification necessary to a successful student. No dentist should take into his office, or encourage a student until he has made his fitness an especial study.

This too prevalent and pitiable practice of taking mere boys into the office, before they have had any previous education, and expecting them to make, honorable, worthy and successful members of the profession is much to be deplored, no business man would take into his employ a book-keeper, on the simple recommendation of his proficiency in penmanship. No more would you expect a youth to make a profound and intelligent lawyer because he possessed rare powers of elocution. It will be admitted by all that the better primary education one may possess, the better will he be prepared to enter upon the study of any specialty he may choose to engage in.

Preceptors should not be satisfied to impart only such knowledge as they themselves possess, but should stimulate and encourage in the minds of their students an earnest and heartfelt desire to emulate the example of those to whom we owe so large a debt of gratitude for the life-long labors they have devoted to scientific research. Do we not all, at times, regret that our early teaching was not more complete, and are we not ready to exclaim with Solomon of old? "O Lord, give me wisdom and knowledge." I would that every student in dentistry was required to graduate from a medical college before he should be deemed qualified to matriculate in a dental college, and I am fully convinced that the time is not very far distant when every dentist will be a physician, not when every physician will be a dentist; for will it not be acknowledged by all that dentistry is but an advance specialty of medicine, and consequently will be best practiced by him who has most knowledge of general medicine, other things being equal. How often is the dentist called upon to anæsthetize a person of whose health he is not at all advised, and if he has no knowledge of the functions of the circulatory system and action of the respiratory organs, ought he be allowed to tamper with life as he must do, unless he calls to his aid medical attendance, which the average dentist is not at all apt to do? How, I ask, can a dentist relieve a case of facial neuralgia, who has no knowledge of nerve connection, of the arrangement of plexuses and ganglions. And yet how often are these very cases thrown in our way, and would, and should be much oftener than they are if we but had the skill to properly diagnose and relieve them.

This advance step may seem to some to be in the far future, if at all, but if you admit the necessity of such an advance, you must certainly be prepared (or prepare your students rather) to receive the reality; for we are living in an age when supply follows closely upon the heels of demand. It may be asked, how is this best to be accomplished, I would answer: First, by a thorough preliminary education; and, second, by matriculating in an institution where all the advantages necessary to the acquirement of such an education can be secured. Dental colleges must either teach a thorough course of medical instruction, or connect themselves with institutions that do. In saying this, I would in no wise cast disparagement upon the good and noble men who have so generously, in many instances, given both time and talent to organize and maintain schools (and good ones too) for dental instruction. To them is due in part the honor of the more than rapid advance made by the profession for the past thirty years, the like of which I believe has never been known, notwithstanding the belief of some, that our profession is numbered among the lost arts. We have as, you well know, been agitating to some extent, for the past five years, in this state, the question of legislative enactment, by which, it is expected to control the acts of the unqualified and unscrupulous in the profession.

If by legislative measures this can be accomplished let us push forward, and never cease our exertions until our demands are granted. But I believed at the time, and do still, that we began at the wrong end of the race, let us bend our energies hard toward the establishment of a school within our own borders, that shall provide the necessary advantages for the enlightenment of those who will embrace them; and then, I believe, we shall experience little or no difficulty in securing such enactment as to bear upon those who do not and will not. And I am sure the right move has already been made in this matter from the encouraging report we get from the board of regents of our university, and more, I am confident by an untiring effort and zeal unabating we shall ere long secure from our legislature the necessary appropriation. And in this I believe we shall have accomplished more in the elevation of the profession, and for the good of the coming generation than by all the legal enactments that could be spread upon our statute books. 'Tis true, we are in receipt of encouraging reports from sister states that are enjoying the benefits of state legislation, and I am only too thankful that there are states, the interests of which are represented by men who are clear headed and intelligent enough to admit of the necessity of such protection as the law may afford. But, O! what a glorious time it will be when it comes, if it ever does, when no need exists for legal enactments. And we can hasten the time by pressing upon the minds of our students the importance of the work, the responsibilities of the work, and of the glorious things yet in store for him who will press forward with an unyielding determination to know all there is within the range of his scientific research and observation. Let us, then, use every means in our power to sink into oblivion the too prevalent idea that dentists, like wax figures, can be made in from three to six months. And still another thing quite as essential is the education of the people, to enable them to discriminate between worthy men and charlatans. This subject is often discussed both in and out of the various societies, but, as yet, there has been very little practical application of what we know the people are most in need, and that is the general distribution of the small, but,—nevertheless, all important—things that the intelligent practitioner is often times apt to neglect, because he thinks every person is acquainted with them. So far as I am able to judge, there is no subject connected with the personal walfare of the people of which they know so little as the proper care and preservation of the teeth.

At previous meetings of this association, as well as at others of like character, I have advocated the matter of conferring with the various publishers of school books for the purpose of securing their approval and consent to the introduction of short, terse, but plain articles, setting forth the advantages to be derived from an early and constant care of the teeth, through the diet and otherwise. I believe that through this great good would, in due time, be accomplished. We should reach many who could never be reached through the usual channels. I have very little knowledge of the matter our modern school books contain, but we can all remember many of the foolish and exciting fables we were treated to in our boyhood days; we all know, too, that the things pressed upon us in early life are more enduring than those received later; and I hope this association will not adjourn until some practical move has been made in this direction.

I would also call your attention to the resolution of 1872, relative to the compilation and publishing of a small manual of dental information, as emanating from this association, for general distribution among our patients, for which a portion of the matter has been ready for more than eight months.

But, gentlemen, there ought and must be more than three or four in this association with willing hearts and ready hands to further this, as well as every other laudable movement connected with the profession. Let us not only pass resolutions, but be as ready to fulfill the duties imposed upon us, regarding them as imperative, though it may require time which we feel we can scarcely give; remember that sacrifices must be made, and each one should be willing to do his share in promoting the general good and bringing nearer that coming day when excavators and drills shall no longer be needed.

